# Intracellular Imaging with Genetically Encoded RNA-Based Molecular Sensors

**DOI:** 10.3390/nano9020233

**Published:** 2019-02-08

**Authors:** Zhining Sun, Tony Nguyen, Kathleen McAuliffe, Mingxu You

**Affiliations:** Department of Chemistry, University of Massachusetts Amherst, Amherst, MA 01003, USA; zhiningsun@umass.edu (Z.S.); tonnguyen@umass.edu (T.N.); kmmcauliffe@umass.edu (K.M.)

**Keywords:** RNA aptamers, biosensors, live-cell imaging, fluorogenic RNA, riboswitch, ribozyme

## Abstract

Genetically encodable sensors have been widely used in the detection of intracellular molecules ranging from metal ions and metabolites to nucleic acids and proteins. These biosensors are capable of monitoring in real-time the cellular levels, locations, and cell-to-cell variations of the target compounds in living systems. Traditionally, the majority of these sensors have been developed based on fluorescent proteins. As an exciting alternative, genetically encoded RNA-based molecular sensors (GERMS) have emerged over the past few years for the intracellular imaging and detection of various biological targets. In view of their ability for the general detection of a wide range of target analytes, and the modular and simple design principle, GERMS are becoming a popular choice for intracellular analysis. In this review, we summarize different design principles of GERMS based on various RNA recognition modules, transducer modules, and reporting systems. Some recent advances in the application of GERMS for intracellular imaging are also discussed. With further improvement in biostability, sensitivity, and robustness, GERMS can potentially be widely used in cell biology and biotechnology.

## 1. Introduction

The detection and quantification of cellular proteins, nucleic acids, and metabolites is critical in understanding cellular signaling pathways and many other physiological processes. These cellular molecules are tightly regulated in living systems. Both their cellular levels and distributions play essential roles for their biological functions. As a result, the development of sensors to characterize the spatial and temporal distributions of cellular targets and to accurately quantify their cellular levels has been a major focus in current biochemical studies [1,2,3].

Although the expression levels of many biomolecules can be measured using traditional methods such as gel electrophoresis, mass spectrometry, liquid chromatography, and NMR spectroscopy [4], most of these techniques require complex pre- and post-treatments on cells and can only deal with cell lysates. These in vitro assays provide limited information on the cellular distributions, live-cell dynamics, or cell-to-cell variations of the target analytes.

Fluorescence imaging, on the other hand, overcomes most of these challenges [5,6,7]. Synthetic fluorescent compounds, such as fluorescein, rhodamine, BODIPY, and cyanine, have been widely used as reporters in developing small-molecule sensors for cellular imaging [8,9,10,11,12,13,14,15,16]. However, the limited biocompatibilities, cellular interferences, and cellular distributions of these non-natural compounds remain major issues that limit their actual biological applications [17,18,19].

Sensors based on naturally occurring proteins or RNA molecules could potentially address these issues in cellular analysis. For example, fluorescent protein (FP)-based sensors were developed soon after the isolation of green fluorescent protein (GFP) from the luminous organ of the jellyfish *Aequorea victoria* [20,21]. FP-based Förster resonance energy transfer (FRET) sensors have advanced the field of bioimaging by quantitatively detecting various classes of targets in living systems [22,23,24,25,26]. However, many critical cellular targets cannot be feasibly detected using these protein-based sensors. This fact is largely due to the limited choice of protein domains that can selectively bind to the target molecules, which should also induce conformational changes that lead to significant FRET changes. Furthermore, the detection range and the signal-to-noise ratio of many FP-based sensors are not ideal for the cellular imaging and detection of target biomolecules [27,28].

Recently, an alternative class of RNA-based fluorescent biosensors has been developed for intracellular applications [29,30,31,32]. In general, these Genetically Encoded RNA-based Molecular Sensors (GERMS) consist of three components: a recognition module, a reporting system, and a transducer module. The recognition module, such as an RNA aptamer (RNA aptamers will be described in more depth in Section 4.1), is an RNA sequence that can specifically recognize target molecules and bind to them with a high affinity [33,34]. The reporting system is normally a fluorescent protein or a fluorogenic RNA aptamer that can bind and induce the fluorescence of its cognate small-molecule dye [35,36]. The transducer module is used to connect the recognition module and the reporting system. These transducers act as switches that can convert target binding events into detectable signals [37].

These novel RNA-based sensors can be genetically encoded and transcribed by cells on their own for long-term studies. GERMS can be easily and rationally modified for the detection of a wide range of target molecules with good selectivity and sensitivity. These genetically encodable sensors have shown promising potential in detecting intracellular RNAs, proteins, metabolites, signaling molecules, and metal ions [29,30,32,38,39,40,41]. GERMS have started to be used to monitor cellular signaling pathways as well as other biological processes [41,42]. There are several great reviews and articles about the design and application of RNA-based nanodevices [43,44,45,46,47,48,49]. In this review, we will focus on a specific emerging group of RNA devices that can be genetically encoded for the intracellular detection of biological analytes. We will first illustrate how to design and engineer the three components of GERMS: the recognition module, transducer module, and reporting system. Recent examples will be further provided to demonstrate the intracellular applications of these novel RNA-based sensors.

## 2. Transducer Modules in GERMS

Because GERMS are used to sense essential biomolecules in live cells, a fundamental question arises: How do GERMS recognize the target molecules and then provide a corresponding signal? The transducer module couples the recognition module with the reporting system in order to realize the entire sensing process. These RNA-based transducers provide an additional layer of modulation to permit an efficient signal transmission. In this section, we will discuss existing transducer modules in the design of GERMS.

### 2.1. RNA Duplex Formation or Helix Slipping

**Figure 1:**
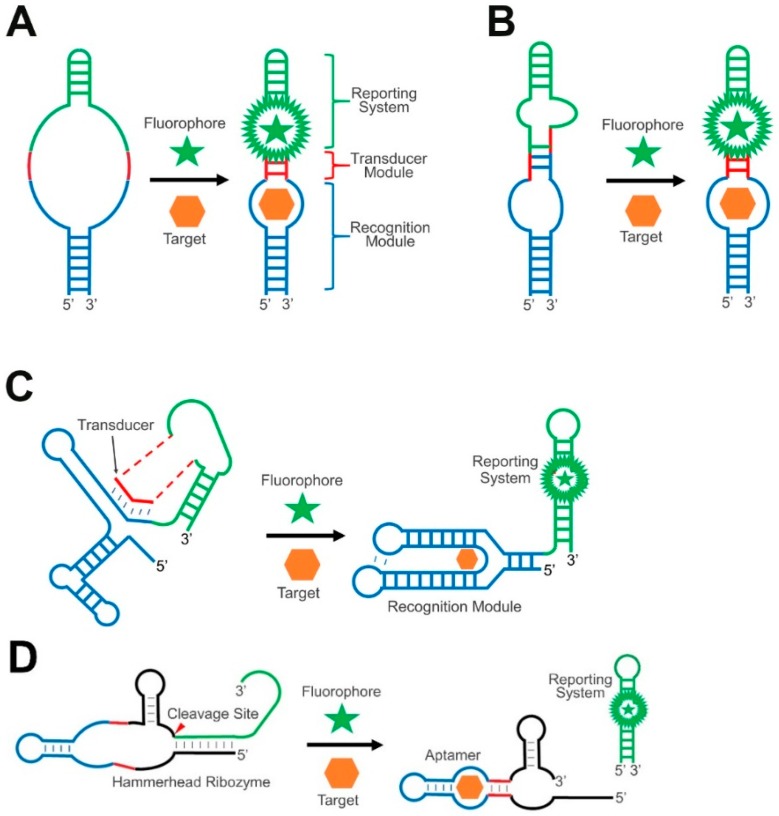
Schematics of different types of transducer module in GERMS. (**A**) The target binding-induced proper folding of the recognition module (blue) can trigger the formation of RNA duplexes (red), which further activate the reporting system (green). (**B**) The target binding induces helix slipping in the transducer module (red) to generate the signal. (**C**) Similar to natural riboswitches, the target binding induces the strand displacement of the transducer sequence (red) from the recognition module (blue) to the reporting system (green). (**D**) The target binding induces the folding (red) and activation of a hammerhead ribozyme to induce the catalytic cleavage and activation of the reporting system (green).

In the general design of GERMS, target binding to the recognition module triggers a conformational change in the transducer module, adjusting the activity of the reporting system. One of the most straightforward conformational changes in RNA devices is the folding and unfolding of a duplex structure (Figure 1A). A duplex formation based on the Watson-Crick or wobble base pairs can be rationally designed as the bridge between the recognition module and the reporting system. Indeed, as demonstrated in the crystal structures of several naturally occurring riboswitches, the most common target binding-induced RNA structural changes are the formation of new duplex regions or the disruption of existing duplexes [50]. In addition, the folding and activation of many reporting systems in GERMS, such as the fluorogenic RNAs, ribosomal binding sites, and transcriptional activators, can also be tuned merely by the formation of a duplex. As a result, duplex formation is one of the most popular and powerful transducer modules in developing allosterically controlled RNA devices, including GERMS.

Helix slipping is another strategy to regulate the formation of the duplex. Helix slipping is a local nucleotide shift in the transducer region. Here, target binding induces a structural change in the recognition module, leading normally to shifts in only one or two nucleotides in the transducer helix, which further activates the reporting system, such as a ribosomal binding site (Figure 1B). The rationale behind this helix slipping principle is that even in the absence of a target, the transducer module should preferably still form a structure, instead of a free form, to better inhibit the activity of the reporting system. As a result, a large signal-to-noise ratio will be realized after the slipping. 

### 2.2. RNA Strand Displacement

The structural rearrangement of the transducer module can also be realized through a strand displacement reaction. Strand displacement-based RNA signal transductions have been widely used in natural riboswitches. Riboswitches are regions in mRNAs that contain a specific evolutionarily conserved target-binding aptamer domain and an expression platform that enables the regulation of the downstream transcription or translation. The competitive binding of a transducer sequence to either a switching sequence in the aptamer domain or the expression platform is critical for the function of riboswitches (Figure 1C). For example, in a naturally abundant thiamine pyrophosphate (TPP) riboswitch, the addition of TPP allows for the formation of a TPP-binding pocket in the aptamer domain, which displaces the transducer sequence, further allowing the formation of an expression platform duplex to inhibit the translation [51].

In general, target binding with riboswitches will alter the relative stability or accessibility of the RNA duplex involved in the displacement reaction. As a result, new thermodynamically more stable duplexes will replace the previously favorable conformations. If the newly formed structure can induce the activation of a reporting system, such strand displacement reactions can be used to engineer RNA-based sensors.

Inspired by the mechanism of these naturally evolved riboswitches, synthetic riboswitches have been engineered into biosensors to detect different biological targets. Here, artificial aptamer domains and synthetic expression platforms are conjugated based on a strand displacement reaction. Computational predictions of the RNA folding and energy landscapes are often used in the generation of these synthetic biosensors. For example, an automated design model has been engineered to generate synthetic riboswitches from aptamers that can activate the translation initiation by up to 383-fold [52]. Statistical thermodynamics models have been made to measure the sequence-structure-function relationships to convert synthetic RNA aptamers into translational regulating riboswitches [53]. There are several factors determining the efficiency of such synthetic riboswitches, including their target-binding affinities, overall induced conformational changes, target and RNA expression levels, interactions with ribosomes and other protein/RNA complexes, as well as the macromolecular crowding effect. Due to the existence of these complex factors, the intracellular and in vivo behaviors of many synthetic riboswitches are still not easily predictable. In situ experimental optimizations are often necessary. It is expected that the further development of advanced computational tools and simplified high-throughput in vivo screening approaches will dramatically improve the performance of these synthetic riboswitch tools.

### 2.3. Ribozyme-Based Transducers

The transducer modules of GERMS can also stem from catalytic cleavage functions, as shown in naturally occurring RNA ribozymes. For example, the hammerhead ribozyme is the most widely studied natural catalytic RNA for this purpose [54,55,56]. The minimal catalytic domain of a hammerhead ribozyme comprises three duplexed stem regions. The proper folding of all these three regions is required for the catalytic self-cleavage of the hammerhead ribozyme. By fusing a target-binding recognition module and a reporting system into two of the three stem regions, the hammerhead ribozyme can function as a transducer in developing RNA-based sensors (Figure 1D). Here, a target-bound recognition module activates the ribozyme so that it self-cleaves and releases the reporting system from the original connection. As a result, biological analytes can allosterically regulate the reporting system in a highly modular pattern. The structure and function of hammerhead ribozymes have been well characterized with rapid kinetics, simple design, and small sizes [57]. Ribozyme-based transducers have been engineered for the in vitro and intracellular measurement of many metabolites [54,58], as well as for intracellular gene regulation [55,56].

In addition to hammerhead ribozymes, several other ribozymes have been identified as potential platforms for engineering the transducer modules. Most of these ribozymes, including twister ribozymes, twister sister ribozymes, pistol ribozymes, Varkud satellite ribozymes, and hairpin ribozymes [59,60,61,62], are known as “small self-cleaving ribozymes” ranging between 50 and 150 nucleotides in length [63]. Having been evolved directly in the living system, these ribozyme scaffolds will likely still function properly after incorporation into genetically encoded RNA devices. The diverse choice and advantageous small sizes of these ribozyme units can be potentially useful for the generation of versatile GERMS, and in the detection of a wide range of cellular targets.

## 3. Reporting Systems in GERMS

### 3.1. Protein-Based Reporters

As mentioned above, fluorescent protein-based sensors have revolutionized cellular imaging. Fluorescent proteins like GFP have been widely used as genetically encodable tags that can be fused to virtually any protein molecules. Various fluorescent proteins with optimized physical and optical properties have been evolved, providing a rich toolbox to study cell biology. Fluorescent protein-based reporting systems are straightforward choices in engineering RNA-based genetic devices. Similar to that shown in synthetic riboswitches, the target recognition module and transducer module can be inserted into transcripts encoding fluorescent proteins. As a result, variations in the cellular target levels will lead to changes in the cellular fluorescence.

Luciferase-induced luminescence signals have also been used to report the efficiency of RNA-based devices. Luciferase is a class of enzymes that can emit light by oxidizing their small-molecule luciferin substrates. Without the light excitement that induces cellular auto-fluorescence, the luciferase-based reporting system can provide a better signal-to-noise ratio than that of fluorescent protein reporters. Furthermore, luciferase signals can be easily quantified [64]. Being widely used for in vitro analysis, the intracellular functions of these bioluminescent systems can be hindered by their overall dim signals, limited choice of wavelengths, and due to the limited availability of the luciferin substrates [65].

To realize such an RNA-based regulation of the protein expression, the RNA sensors normally function at the cotranscriptional level (by alternating RNA splicing or intron synthesis) [66,67,68], the post-transcriptional level (by regulating mRNA stability or availability) [69,70,71,72,73], or the translational level (by controlling the initiation, termination, and specificity of translation) [74,75,76,77]. In addition to the relatively low signal-to-noise ratio, one major challenge is the limited temporal resolution of such fluorescent protein or luciferase-based reporters. This is mainly due to the time required for the translation and for nascent fluorescent proteins or enzymes to mature into their activated forms.

### 3.2. Fluorogenic RNA Complexes

**Figure 2:**
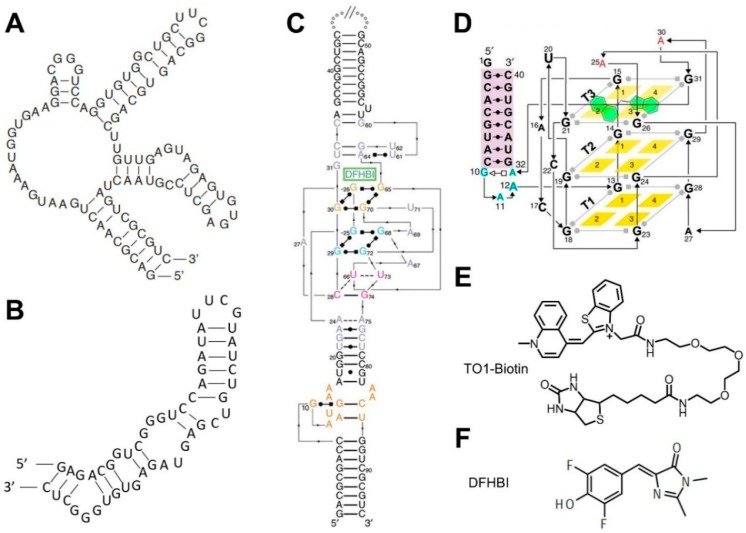
2D/3D structures of fluorogenic aptamers and the chemical structures of their ligands. (**A**) Spinach aptamer 2D structure. (**B**) Broccoli aptamer 2D structure. (**C**) Spinach aptamer 3D structure [88]. (**D**) Mango aptamer 3D structure [85]. (**E**) Mango aptamer’s cognate dye TO1-Biotin. (**F**) Spinach and Broccoli aptamers’ cognate dye DFHBI.

Fluorogenic RNA complexes are composed of a fluorogenic aptamer and a small-molecule chromophore that exhibit fluorescence when bound together [78]. A fluorogenic RNA aptamer is a short nucleic acid strand that can specifically bind to and activate the fluorescence of its corresponding chromophore. For example, Spinach (Figure 2A,C), an RNA mimic of GFP, is one of the most popular fluorogenic aptamers in developing GERMS [35]. Spinach binds to a DFHBI chromophore and turns on its fluorescence. DFHBI (Figure 2F) is cell membrane permeable and has a low cellular background signal. After genetically conjugating Spinach to the target RNA molecules, DFHBI can be added externally to track the cellular locations and concentrations of the RNA targets.

Spinach can also be engineered as the reporter for the detection of metabolites and proteins. There is a sequence-independent stem region in Spinach that plays an important structural role in the activation of the DFHBI fluorescence [29]. By fusing a target-binding aptamer and a transducer module into this stem region, the binding of the target will fold the aptamer and subsequently stabilize the stem of Spinach to exhibit fluorescence [29]. It is critical that the target-binding aptamers should be unstructured until they are bound to the target. These Spinach-based RNA sensors can be used to detect concentration variations of the targets in real time both in vitro and in living cells [79].

To improve the folding of Spinach, the systematic mutagenesis of the original Spinach RNA has led to the development of Spinach2 [80]. Spinach2 exhibits a brighter fluorescence and increased thermal stability than Spinach in living cells [80]. Another notable Spinach derivative is named Baby Spinach [81]. The shortened sequence of Baby Spinach reduces the overall size of the Spinach tag, which may allow for the incorporation of multiple fluorogenic RNAs for cellular tracking, and which may increase the cellular biostability of these fluorogenic RNA complexes [81].

To improve the intracellular folding and brightness of these fluorogenic RNA complexes, fluorescence-activated cell sorting (FACS) has been used to identify Broccoli (Figure 2B) [79]. Broccoli is a short sequence, which shows increased folding and fluorescence in cells, even at low magnesium levels, making it a suitable option for live-cell imaging. A particularly useful version of Broccoli in engineering biosensors is called Split-Broccoli, where the Broccoli fluorescence is activated only upon the reassembly of two split pieces of Broccoli RNA [82]. For example, Split-Broccoli can be used to visualize intracellular RNA-RNA hybridizations with faster kinetics than fluorescent protein-based reporters [82].

Another recent advance in these fluorogenic RNA complexes is the Corn/DFHO system [83]. This complex is unique in its high photostability and red-shifted fluorescence emission. For example, in imaging the activities of RNA Polymerase III in live mammalian cells, a better performance has been demonstrated in Corn than in Broccoli [83].

Similarly, several other fluorogenic RNA complexes have been developed as potential reporting systems in engineering GERMS. For example, RNA Mango (Figure 2D) is a class of fluorogenic RNA aptamers that exhibits a bright fluorescence when bound to TO1-biotin (Figure 2E) or TO3-biotin dyes [84]. Mutagenesis of the original Mango I aptamer has resulted in the generation of Mango II, Mango III, and Mango IV aptamers with optimized fluorescence intensities, chromophore-binding affinities, and a salt dependency [85]. In another example, the binding of a DNB or SRB-2 aptamer can separate and activate the fluorescence of a sulforhodamine-dinitroaniline (SR-DN) dye-quencher pair, exhibiting a bright orange/red fluorescence for intracellular imaging [86,87].

Table 1 summarizes the commonly used fluorogenic RNA complexes that can be used as potential reporters for the sensor development [89]. Right now, these fluorogenic RNA complexes are still far less versatile than the existing fluorescent protein toolbox. However, with the rapid development of brighter, more photostable, and multi-color fluorogenic RNA complexes, we expect that more sensitive, multiplexed, and quantitative imaging can be achieved by these direct RNA-based reporting systems.

## 4. Recognition Modules in GERMS

The target-specific recognition module is another critical unit in GERMS. In general, to detect cellular nucleic acid targets, RNA strands with complementary sequences can be directly used as highly specific recognition modules. On the other hand, for most non-nucleic acid targets, RNA aptamers can be engineered as the recognition modules in GERMS.

### 4.1. Aptamers and Conventional SELEX

Aptamers, first reported in 1990 [90,91], are oligonucleotide strands that have a high binding affinity and specificity toward their targets. Aptamers can be comparable with antibodies in many ways. Aptamers can be either selected from a large random library pool using Systematic Evolution of Ligands by EXponential enrichment (SELEX) or directly adapted from naturally existing riboswitches [90,91,92,93,94,95]. Depending on the sequence, RNA aptamers can form diverse and intricate three-dimensional structures, allowing them to tightly and specifically bind with various biological targets.

SELEX has been widely used in aptamer selection. In general, SELEX begins with a chemically synthesized DNA library. The library contains numerous (normally 10^14^–10^15^) oligonucleotides with a random sequence in the same region, which is flanked by known fixed sequences. After the PCR and in vitro transcription of the synthetic DNA library into an RNA library, several selection steps are introduced to remove unwanted unbound oligonucleotides. The RNA sequences that are bound to the target are then released and reverse transcribed into DNA, before being further amplified by PCR. Such multiplied DNA molecules are then transcribed, in vitro, back into RNA, and a new selection round begins. Up to 20 selection rounds are usually performed in conventional SELEX to enrich aptamers with a high target binding affinity. Negative and counter SELEX are often processed at the same time to ensure a selective binding toward the target [96].

Using SELEX, RNA aptamers have been identified toward various targets, ranging from metal ions (e.g., Co^2+^) [97], small organic molecules (e.g., amino acids [98], ATP [99], antibiotics [100], vitamins [101], and organic dyes [102]), to proteins (e.g., thrombin [103], transcription factors [104], and HIV-associated proteins [105]), and even to entire cells or microorganisms (e.g., virus and bacteria [106]). Through the SELEX procedure, RNA aptamers can be generated toward essentially almost any type of biomolecule.

### 4.2. Advanced SELEX Approaches for GERMS

In addition to the conventional SELEX procedure, several other advanced SELEX methods have been developed that are particularly suitable for engineering GERMS. Among others, three notable methods are Capture-SELEX, ribozyme-based SELEX, and graphene oxide-based SELEX.

**Figure 3:**
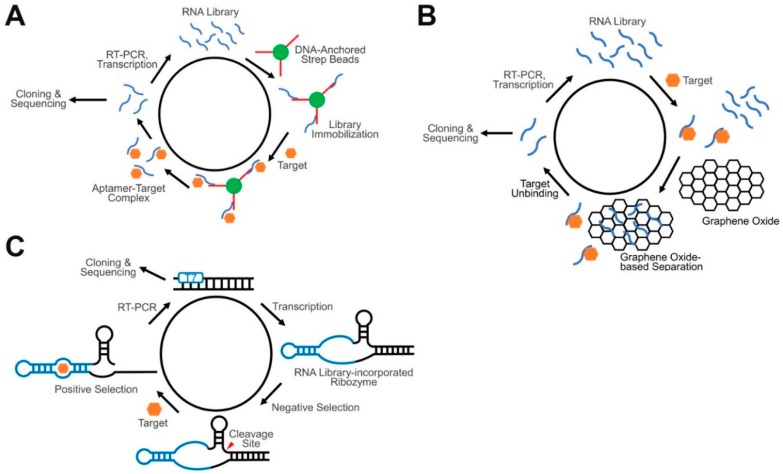
Schematics of advanced SELEX approaches for GERMS. (**A**) In a Capture-SELEX, an RNA library is immobilized on the surface of beads via the attached short capture DNA. RNA aptamers that can bind with the target and undergo conformational changes will be eluted and further amplified. (**B**) In a graphene oxide SELEX, target unbound RNA strands adsorb onto the surface of graphene oxide and separate from target-binding aptamers. (**C**) In a ribozyme-based SELEX, target-binding aptamers induce the catalytic self-cleavage of the ribozyme. Based on the band shift in gel electrophoresis, aptamer-containing constructs can be isolated from the RNA pool.

Capture-SELEX is different from conventional SELEX in that it does not require the immobilization of the target compounds to beads or surfaces [107]. In a regular Capture-SELEX method (Figure 3A), short capture DNA strands are first attached to the surface of magnetic beads, and then an oligonucleotide library is immobilized to the beads by binding to the capture strands through the fixed sequence region in each oligonucleotide. By adding a solution of the solvated target, aptamers that can bind to the target and undergo conformational changes to displace the capture strands are then eluted for further enrichment. This method opens opportunities for RNA aptamer selection against target molecules that cannot be easily immobilized or chemically modified, such as several small metabolites and signaling molecules. In addition, similar to riboswitches, the identified aptamers in the Capture-SELEX have been already optimized to respond to target binding by changing the RNA conformation, which is important for sensor development. Instead of merely screening for the recognition module, Capture-SELEX allows the direct identification of both the recognition module and the transducer module for the development of GERMS.

Ribozyme-based SELEX is another powerful method for developing RNA-based sensors. As mentioned above, ribozymes are naturally occurring RNA strands that can catalytically trans- or cis-cleave at a particular position or sequence [108]. One potential challenge in performing small molecule-targeting by SELEX is that these small targets normally cannot lead to significant conformational or property changes between the bound complexes and the free aptamers. Ribozyme-based SELEX, however, utilizes the self-cleaving properties of ribozymes to realize massive target-induced size changes in the RNA strands. To design an oligonucleotide library for such a purpose, a random RNA region is inserted into a structurally critical domain of the ribozymes, such as one of the three stem regions in a hammerhead ribozyme [109] (Figure 3C). Ribozyme-based SELEX starts with a negative selection, in the absence of the targets, to remove autonomously self-cleaved RNA strands. Uncleaved aptamers are then PAGE gel-purified and incubated with targets in the positive selection. During this step, the cleaved RNA strands are isolated via gel-purification, further reversibly transcribed, amplified by PCR, and transcribed back into full-length RNA strands for the next round of selection. The hammerhead ribozyme is the most widely used ribozyme in this type of SELEX. The identified aptamers can selectively bind with the target and further induce the folding of a stem region (i.e., the transducer) of the hammerhead ribozyme. Again, both the recognition module and the transducer module can be directly identified for the development of GERMS. In addition, with the diverse choices of different classes of naturally occurring ribozymes, various target molecules can potentially be recognized with different signal transduction mechanisms.

Graphene oxide (GO) is another platform which has recently become popular in screening for aptamers. GO-SELEX is based on the non-specific adsorption of the oligonucleotide library by graphene oxide [110]. The library is normally pre-incubated with the target, after which GO is added. Single-stranded oligonucleotides can be adsorbed by GO due to π–π stacking, while target-bound complexes remain free in the solution. After removing sequences not bound to GO through centrifugation, the target-bound oligonucleotides are then separated and amplified by reverse transcription, PCR and transcription (Figure 3B). GO-SELEX also does not require a target immobilization. The selected aptamers have also been optimized in order to obtain the property of target-induced conformational changes. GO-SELEX is a simple, high-speed, high-throughput aptamer screening method that can be applied to various target molecules [111].

### 4.3. Riboswitch-Based Recognition Modules

Riboswitches are naturally occurring recognition modules for many critical cellular metabolites and signaling molecules [112,113]. Another way of developing GERMS is by directly adopting these riboswitches as recognition modules. As mentioned previously in this manuscript, a riboswitch consists of an aptamer domain, a switching sequence, and an expression platform. The aptamer domains in the riboswitches have been naturally evolved to selectively bind with various cellular targets including enzyme cofactors, nucleotide precursors, amino acids and atomic ions [114]. For example, the *metH* S-adenosylhomocysteine (SAH) riboswitch can selectively recognize SAH in preference to S-adenosylmethionine (SAM) by 1000-fold [115], while SAM and SAH differ only by a methyl group. As a result, these SAH riboswitches have been used to develop sensors to measure SAH levels as well as the methyltransferase activities in vitro, which further facilitates the screening of novel MTase inhibitors [116].

During the conventional in vitro SELEX process, it is difficult to perform negative or counter SELEX against all the diverse and structurally related molecules in the cell. In addition, obtaining aptamers that have a suitable target-binding affinity is in many cases still a challenge. Most in vitro identified aptamers should be further tested and optimized in the real cellular environment. The major advantage of riboswitches over SELEX-generated aptamers is that riboswitches have been evolved to have the type of in vivo selectivity and binding affinity needed to recognize cellular targets. 

### 4.4. Specific Base Pair Formation

RNA-based recognition modules can also be designed based on sequence-specific base pairings. In addition to the traditional Watson-Crick (A to U and C to G) base pairs, wobble base pairs (e.g., G to U or I to C), G-quadruplexes, and metallo-base pairs can also be engineered as specific recognition modules for the development of RNA-based sensors. For example, we have recently developed a C–Ag^+^–C metallo-base pair-based fluorogenic RNA sensor for the intracellular imaging of Ag^+^ ions [117]. In this study, these metallo base pairs can function as both the recognition module and the transducer module. The signal transduction mechanism is similar to the one discussed above in Section 2.2.

## 5. Recent Examples of GERMS 

GERMS have been successfully applied in multiple intracellular studies. For example, the Jaffrey lab developed a type of allosteric Spinach sensor. Similar to the one shown in Figure 1A, the allosteric Spinach sensor comprises a target-binding aptamer (recognition module), a transducer duplex (transducer module), and a Spinach aptamer (reporting system). This type of sensor has been engineered to detect diverse metabolites and proteins, such as adenosine diphosphate, SAM, guanosine triphosphate, thrombin, and MCP coat protein [29,30]. In Table 2, we have shown some of the existing GERMS for intracellular applications. The optimal sensors normally exhibited 10- to 40-fold increases in fluorescence upon binding their cognate ligands. Notably, a SAM-targeting allosteric Spinach sensor has been used to reveal cell-to-cell variations in the SAM metabolism, which cannot be observed via conventional methods [29].

We previously engineered Spinach riboswitches, nature-inspired GERMS for detecting metabolites in the cytosol of cells with high target selectivity. For example, by engineering the Spinach aptamer into the expression platform in a natural *thiM* TPP riboswitch, we developed TPP-targeting GERMS. Similar to that shown in Figure 1C, the TPP-dependent natural switching mechanism of the riboswitch enables the proper folding of the Spinach aptamer, which then activates the fluorescence of DFHBI [32]. Compared to aptamers selected by in vitro SELEX, naturally occurring riboswitches have inherent advantages in their high affinity and selectivity for cellular targets. Currently, many naturally occurring riboswitches have been discovered, and the Spinach riboswitch strategy enables the direct conversion of riboswitches into functional GERMS. 

We recently engineered another class of RNA-based fluorescent sensors, termed RNA integrators, for the intracellular detection of low-abundance metabolites [132]. In this design, the self-cleaving property of hammerhead ribozymes is used to activate the Broccoli aptamer upon binding to target molecules. Similar to that shown in Figure 1D, in the presence of target molecules, the recognition module rearranges to form the binding pocket, which leads to the formation of the catalytic pocket in the hammerhead ribozyme. As a result, target binding induces the activation of the self-cleavage of the ribozyme and releases the downstream Broccoli aptamer sequence, which then binds DFHBI in order to emit fluorescence. Here, each target molecule can induce the cleavage of multiple copies of the RNA integrator, resulting in an amplified signal.

**Figure 4:**
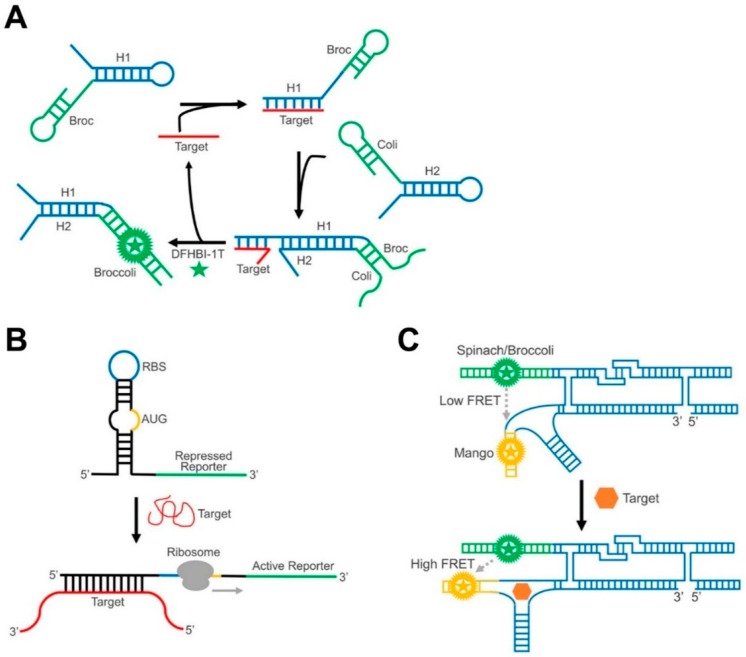
Schematics of RNA nanotechnology-inspired GERMS. (**A**) In a CHARGE circuit, target binding (red) induces the catalytic hybridization of multiple hairpin assemblies (blue), further activating an amplified signal from reassembled Broccoli RNA (green). (**B**) In a toehold switch sensor, target binding releases the ribosome binding site (RBS) and a start codon (AUG), which activates the expression of the reporting system (green). (**C**) In an RNA origami construct, target-induced structure change can regulate the distance and FRET efficiency between two fluorogenic RNA complexes.

In addition to these nature-inspired designs, GERMS can also be engineered based on recent advancements in DNA and RNA nanotechnology. For example, our lab has recently developed the first GERMS based on an RNA logic circuit, termed the Catalytic Hairpin Assembly RNA circuit, that is Genetically Encoded (CHARGE) [131]. In our CHARGE sensor design, two complementary RNA hairpins stay separate from each other in the absence of a target (Figure 4A). After adding the target, one hairpin opens based on a toehold-mediated strand displacement reaction, and then induces the subsequent hybridization of both hairpins [133]. The target can then be recycled to trigger the hybridization of multiple copies of hairpins. By coupling with split-Broccoli, we were able to image cellular RNA targets with a high sensitivity [131].

In another example showing that dynamic RNA nanotechnology can contribute to the design of GERMS, the Yin group has developed toehold switches to detect target RNAs with an average ON/OFF ratio of over 400 [134]. The toehold riboswitch functions by the target-induced post-transcriptional activation of the gene expression (Figure 4B). Taking advantage of toehold-mediated linear-linear interactions [133], target RNA can bind with the sequences around the ribosome binding sites (RBS) and a start codon (AUG), triggering a branch migration process to expose the RBS and the start codon. As a result, the presence of the target RNA strand initiates the translation of the downstream fluorescent protein and emits a corresponding fluorescence signal. The orthogonality and programmability of toehold switches can even allow for the independent regulation of 12 genes and is used to construct complex genetic circuits [134].

Synthetic RNA nanotechnology, i.e., the design and construction of artificial RNA nanostructures, can also provide a useful scaffold to improve the performance of GERMS. For example, the Andersen group has recently reported a single-stranded RNA origami FRET system [46]. In their nanoconstruct, two fluorogenic RNA aptamers, Spinach and Mango, were placed in close proximity following a designed pattern (Figure 4C). In the absence of target molecules, the Spinach and Mango pair produced a limited FRET signal. Upon target binding, the origami rearranged the structure, bringing the two aptamers closer to each other and producing a large FRET signal. This construct has been successfully genetically encoded in *E. coli* cells, demonstrating its potential for intracellular imaging.

Other than the examples described above, GERMS can also function, in a way, as logic gates. Alam et al. showed that Split Broccoli aptamers can be converted into an AND gate for monitoring the assembly of RNA–RNA hybrids [82]. The Khisamutdinov group has recently demonstrated a new generation of smart RNA nanodevices based on RNA aptamers [129]. In their approach, the Malachite Green aptamer and the Broccoli aptamer were engineered into four types of oligonucleotide-responsive RNA logic gates (AND, OR, NAND and NOR), which offer a new route to engineer “label-free” ligand-sensing regulatory circuits and nucleic acid detection systems.

## 6. Conclusions and Outlook

Over the past few years, GERMS have emerged for live-cell imaging and the detection of various RNAs, proteins, metabolites, synthetic compounds, and ions. The high versatility of these RNA nanostructures has provided GERMS with a wide choice regarding the recognition modules, transducer modules, and the reporting systems. GERMS can be developed toward various targets with both a high binding affinity and selectivity. The sensitivity, modularity, and dynamic range of these RNA-based sensors have been dramatically improved.

One critical challenge in the rational design of GERMS is to understand how the recognition module changes its conformation after binding to its target. Indeed, it can be difficult to design transducer modules if the structures of both the apo- and holo- forms of the recognition module remain unknown. The crystal structures for most existing riboswitches have been solved. However, for many SELEX-generated aptamers, we still have limited knowledge about their tertiary structures. On the other hand, computational simulations have been used to assist the design and engineering of GERMS. Unfortunately, it is still challenging to accurately simulate many complex intramolecular interactions among different modules within these functional RNA structures, without mentioning the challenge in predicting how target binding can thermodynamically and kinetically change the conformation of GERMS.

Currently, it is still taking a long time and many trials to develop a functional RNA-based sensor. The number of selection rounds will be greatly reduced if there are guidelines for the pairing of different modules in GERMS. In other words, if we could design the transducer module simply by looking at the sequence of the recognition module and its binding pocket, this would greatly improve the design efficiency. Potential milestones in engineering GERMS will likely depend on revolutionary algorithms in computational simulations and a more comprehensive understanding of the relationships between RNA sequences and their corresponding tertiary structures.

Another limitation in applying GERMS for mammalian cells or in vivo imaging is RNA degradation and low-level expression. Short RNA constructs, like those in most GERMS, can be rapidly degraded in eukaryotic cells. One potential solution for improving the expression level of GERMS is based on circular RNA constructs. Circular RNAs have been identified in vivo as naturally evolved stable RNA molecules. Circular RNAs do not have either a free 5’- or 3’-end, which makes them invulnerable to most cellular exonucleases. Recent studies have shown that these circular RNAs can be stable for days-to-weeks and that they accumulate at high levels in diverse eukaryotic organisms [134,135,136,137,138]. The potential incorporation of the circular RNA strategy in GERMS may open a new window for the in vivo imaging and detection of targets that have not been successfully studied with available RNA- or protein-based sensors.

In conclusion, we have summarized in this review the basic design principles and recent applications of GERMS for bioimaging and the detection of cellular targets. The versatility of these RNA-based sensors makes GERMS highly useful for studying essentially any molecule in living cells. GERMS have shown great potential for future live-cell imaging. After improving their biostability, sensitivity, target selectivity, and kinetics, the next steps will likely be the engineering of GERMS into working sensors in eukaryotic cells, as well as the generation of universal protocols for developing GERMS toward any target of interest.

## Figures and Tables

**Table 1:** Commonly used fluorogenic RNA complexes and their spectral properties.

Aptamer	Fluorophore	*K*_D_ (nM)	Ex./Em. (nm)	Ɛ (M^−1^cm^−1^) ^a^	ɸ ^b^	Brightness ^c^
Spinach [35]	DFHBI	540	469/501	24,300	0.72	100
Spinach2 [31]	DFHBI	530	447/501	22,000	0.72	91
Spinach2 [31]	DFHBI-1T	560	482/505	31,000	0.94	167
Spinach2 [31]	DFHBI-2T	1300	500/523	29,000	0.12	20
Broccoli [79]	DFHBI-1T	360	472/507	29,600	0.94	159
Corn [83]	DFHO	70	505/545	29,000	0.25	41
Mango [84]	TO1-Biotin	3.2	510/535	77,500	0.14	62
Mango [84]	TO3-Biotin	5.1	637/658	9300	N/A ^d^	N/A
Mango II [85]	TO1-Biotin	0.7	510/535	77,000	0.2	88
Mango III [85]	TO1-Biotin	5.6	510/535	77,000	0.56	247
Mango IV [85]	TO1-Biotin	11.1	510/535	77,000	0.42	185
DNB [87]	SR-DN	800	572/591	50,250	0.98	282
SRB-2 [86]	SR-DN	1400	579/596	85,200	0.65	317

^a^ Ɛ—Extinction Coefficient. ^b^ ɸ—Quantum Yield. ^c^ Brightness—Extinction Coefficient × Quantum Yield relative to Spinach-DFHBI. ^d^ N/A—Not Available.

**Table 2:** Properties and design of existing GERMS for intracellular applications. ^a^

Target	Recognition Module Source	Transducer Module Type	Reporting System Type	EC_50_ (μM)	ON/OFF ^b^	Cell System
ADP [29]	SELEX	Duplex Formation	Spinach	270	20	Bacteria
5-HTP [118]	SELEX	Duplex Formation	Broccoli	N/A ^c^	>5	Bacteria
L-DOPA [118]	SELEX	Duplex Formation	Broccoli	N/A	>5	Bacteria
MS2 coat protein [30]	SELEX	Duplex Formation	Spinach	~0.6	41.7	Bacteria
MS2 coat protein [119]	SELEX	Ribozyme	BFP	N/A	1.8	Mammalian
Neomycin [120]	SELEX	Ribozyme	β-galactosidase	N/A	25	Yeast
Streptavidin [30]	SELEX	Duplex Formation	Spinach	<0.2	10.3	Bacteria
Tetracycline/Theophylline [121]	SELEX	Ribozyme	EGFP	N/A	N/A	Yeast
Tetracycline [122]	SELEX	Ribozyme	Luciferase/EGFP	35.4	4.8	Mammalian
Theophylline [123]	SELEX	Ribozyme	EGFP	~200	10	Bacteria
c-AMP-GMP [38]	Riboswitch	Duplex Formation	Spinach	4.2	~8 (37 °C)	Bacteria
c-di-AMP [39]	Riboswitch	Strand Displacement	Spinach2	3.4 & 29	2.4 & 9.1	Bacteria
c-di-GMP [38]	Riboswitch	Duplex Formation	Spinach	0.23	~6 (37 °C)	Bacteria
c-di-GMP [124]	Riboswitch	Duplex Formation	Spinach2	0.005–0.4	~6 (37 °C)	Bacteria
c-di-GMP [125]	Riboswitch	Strand Displacement	TurboRFP	N/A	38	Bacteria
Guanine [126]	Riboswitch	Ribozyme	EGFP	N/A	9.6	Mammalian
SAM [29]	Riboswitch	Duplex Formation	Spinach	120	25	Bacteria
TPP [32]	Riboswitch	Strand Displacement	Spinach	9	15.9	Bacteria
TPP [127]	Riboswitch	Strand Displacement	EGFP	N/A	~5	Plant
TPP [128]	Riboswitch	Ribozyme	tRNA	N/A	43	Bacteria
N-peptide [129]	Ribonucleoprotein complexes	Ribozyme	EGFP/SEAP	N/A	~12	Mammalian
RNA [130]	Base Pairing	Ribozyme	EGFP	N/A	~10	Bacteria
RNA [131]	Base Pairing	Strand Displacement	Split Broccoli	~0.001	2.2	Bacteria

^a^ Only one example is given when the same design principle has been used to detect the same target molecules. ^b^ ON/OFF indicates the number of fold enhancements in the *in vitro* fluorescence of GERMS after adding the target (measured at 25 °C unless otherwise stated). ^c^ N/A—Not Available.

## Abbreviations

GERMS	Genetically Encoded RNA-based Molecular Sensors
FRET	Förster Resonance Energy Transfer
FACS	Fluorescence-Activated Cell Sorting
SELEX	Systematic Evolution of Ligands by EXponential enrichment
GO	Graphene Oxide
PCR	Polymerase Chain Reaction
PAGE	Polyacrylamide Gel Electrophoresis
CHARGE	Catalytic Hairpin Assembly RNA circuit that is Genetically Encoded
BODIPY	Boron Dipyrromethene
FP, GFP, EGFP, BFP, RFP	Fluorescent Protein, Green Fluorescent Protein, Enhanced Green Fluorescent Protein, Blue Fluorescent Protein, Red Fluorescent Protein
DFHBI	3,5-difluoro-4-hydroxybenzylidene Imidazolinone
DFHO	3,5-difluoro-4-hydroxybenzylidene Imidazolinone-2-oxime
DNB, SRB, SR-DN	Dinitroaniline-Binding aptamer, Sulforhodamine B, Sulforhodamine-Dinitroaniline
TO-Biotin	Thizole Orange-Biotin
Co^2+^, Ag^+^	Cobalt ion, Silver ion
ATP, ADP, AMP, GMP	Adenosine Triphosphate, Adenosine Diphosphate, Adenosine Monophosphate, Guanosine Monophosphate
HIV	Human Immunodeficiency Virus
SAH, SAM	S-adenosylhomocysteine, S-adenosylmethionine
MTase	Methyltransferase
TPP	Thiamine 5’-pyrophosphate
MCP	MS2 Coat Protein
5-HTP	5-hydroxytryptophan
L-DOPA	L-3,4-dihydroxyphenylalanine
